# Epidemiological Characteristics of COVID-19 during Seven Consecutive Epidemiological Waves (2020–2022) in the North Bačka District, Serbia

**DOI:** 10.3390/v15112221

**Published:** 2023-11-07

**Authors:** Jelena Banjac, Vladimir Vuković, Tatjana Pustahija, Nebojša Bohucki, Dragica Kovačević Berić, Snežana Medić, Vladimir Petrović, Mioljub Ristić

**Affiliations:** 1 Public Health Institute Subotica, 24000 Subotica, Serbia; drbanjac14@gmail.com (J.B.); drbohucki@gmail.com (N.B.); dberic65@yahoo.com (D.K.B.); 2 Institute of Public Health of Vojvodina, 21000 Novi Sad, Serbia; tatjana.pustahija@mf.uns.ac.rs (T.P.); snezana.medic@mf.uns.ac.rs (S.M.); vladimir.petrovic@izjzv.org.rs (V.P.); mioljub.ristic@mf.uns.ac.rs (M.R.); 3 Department of Epidemiology, Faculty of Medicine, University of Novi Sad, 21000 Novi Sad, Serbia

**Keywords:** COVID-19, SARS-CoV-2, epidemiological waves, reinfections, Serbia

## Abstract

The COVID-19 pandemic continues to pose a threat to global public health. The purpose of this research was to determine the epidemiological characteristics of COVID-19 in the North Bačka district while observing seven pandemic waves. The cross-sectional study was based on data from the COVID-19 surveillance database of the Institute for Public Health of Vojvodina during the period from March 2020 to December 2022. A total of 38,685 primary infections and 4067 reinfections caused by SARS-CoV-2 were notified. Pandemic waves caused by the Delta variant (cumulative incidence rate of 2482.37/100,000) and by the Omicron variant (cumulative incidence rate of 2994.45/100,000) emerged as significant focal points during the surveillance period. Over the course of three consecutive years (2020–2022), women were more affected (50.11%, 54.03%, and 55.68%, respectively). The highest incidence rates in age-specific categories were recorded in 2021 for the age group 40–49 (1345.32 per 10,000 inhabitants), while in 2022, they shifted towards the elderly population. Regarding vaccination status at the time of diagnosis, in 2021, around 15% of patients were vaccinated, while in 2022, the number increased to 37%. The most widely received vaccine was BBIBP-CorV (67.45%), followed by BNT162b2 (19.81%), Gam-COVID-Vac (9.31%), and ChAdOx1 nCoV-19 (3.42%) vaccine. The implementation of stringent public health measures and their mitigation, together with the emergence of new variants, influenced the dynamics of COVID-19 pandemic waves in the North Bačka district. Notably, throughout the study period, the working-age population was the most affected, along with females, with a mild clinical presentation dominating. Reinfections were most frequently recorded during the latter pandemic waves. Dealing with this pandemic has provided some valuable lessons for the development of future strategies in the case of a similar public health crisis.

## 1. Introduction

The first cases of COVID-19, caused by the SARS-CoV-2 virus, were initially reported in Wuhan, China, in December 2019 [1]. For most of these cases, there was an epidemiological connection with the Wuhan seafood wholesale market, where, in addition to the sale of seafood, poultry and wild animals were sold as well [2]. While SARS-CoV-2 was isolated from environmental samples of this market, some phylo-epidemiological analyses suggest the possibility that the virus could have been imported from other places [3]. Due to the worldwide spread of the infection, the World Health Organization (WHO) declared a global pandemic in March 2020, initially affecting 114 countries [4].

In the Republic of Serbia, the first case of COVID-19 was confirmed on 6 March 2020 [5]. As of 10 August 2023, a total of 2,545,186 COVID-19 cases have been documented in the Republic of Serbia [6].

SARS-CoV-2 is a positive single-stranded RNA virus classified in the Betacoronavirus genus [7]. It is primarily transmitted through close contact with infected respiratory droplets, which are produced after sneezing, coughing or speaking loudly. However, indirect transmission routes, such as airborne transmission, fomite transmission, and transmission through biological materials, have also been observed [8]. Both asymptomatic and symptomatic patients can spread the infection, and the viral loads are significantly higher in the nasal cavities than in the throat [9].

The spectrum of clinical manifestations of COVID-19 ranges from asymptomatic to critical forms, with a potentially unfavorable outcome [9]. The Centres for Disease Control and Prevention (CDC) indicate that symptoms and signs caused by SARS-CoV-2 may include fever, dry cough, fatigue, myalgia, arthralgia, sore throat, headache, nasal congestion, diarrhea, nausea, vomiting, various skin rashes, dizziness, and loss of the sense of smell and taste [10].

Several risk factors have been identified and refer to the existence of comorbidities (cardiovascular diseases, hypertension, obesity, chronic obstructive pulmonary disease, malignancies and cerebrovascular diseases), older age, as well as male sex [11,12]. However, with the appearance of the Omicron variant, females were more likely to be (re)infected [13,14,15]. Some research suggests that a healthy diet and atopic conditions may play a protective role and thus prevent disease progression and poor clinical outcomes [11].

The main non-pharmacological measures were focused on both group prevention (restricting travel, prohibiting gatherings, closing educational and cultural institutions, and imposing quarantine) and individual actions (wearing protective masks, practicing hand hygiene and physical distancing, and isolating patients at home) [16]. However, the most important preventive measure for controlling COVID-19, particularly in reducing hospitalization and mortality rates, is immunization [17]. The impact of the COVID-19 pandemic has resulted in increased unemployment, an industrial recession, a decline in social activities like entertainment and sports events, and disruptions in the global supply chain [18]. Physical distancing, school closures, travel restrictions, along with extended periods of home isolation certainly had social consequences [19]. Additionally, strict quarantine measures, coupled with economic crises and unemployment, led to various ways of coping with daily stress, which undoubtedly impacted mental health [20].

This research aimed to investigate the epidemiological characteristics of COVID-19 in the North Bačka district, Republic of Serbia, in the period from the first notified case (6 March 2020 to 31 December 2022).

## 2. Materials and Methods

### 2.1. Study Setting

The North Bačka district is located in the north of the Vojvodina province, Republic of Serbia, and it consists of three municipalities—Subotica, which serves as an administrative center, Bačka Topola, and Mali Iđoš. According to the 2022 census, the population of the North Bačka district was 160,163 inhabitants [21].

We have conducted a cross-sectional study on the laboratory-confirmed SARS-CoV-2 positive cases in patients from the North Bačka district notified during seven consecutive pandemic waves, i.e., from 6 March 2020 until 31 December 2022.

### 2.2. Laboratory Procedures

The detection of the SARS-CoV-2 was performed on a sample of the patient’s nasopharyngeal swab. The samples were tested with the reverse-transcription polymerase chain reaction (RT-PCR) method or, from November 2020, with the antigen rapid diagnostic tests (Ag-RDT) when they became widely available in Serbia, as previously described [5]. Due to its high sensitivity and specificity, RT-PCR is held up as the gold standard for diagnosing COVID-19. A negative result of the Ag-RDT accompanied by the symptoms corresponding to COVID-19 did not exclude the existence of the disease, and therefore, it was necessary to perform RT-PCR testing to obtain definitive confirmation of the diagnosis. Thus, a case of COVID-19 was considered when a patient tested positive on a single or repeated RT-PCR or on the Ag-RDT [22].

### 2.3. Data Collection

The patients’ data were collected during the testing through an epidemiological questionnaire providing basic socio-demographic information, present symptoms and signs of the disease together with the date of their onset, and the date, type and result of the laboratory COVID-19 test. Other types of data included present comorbidities, i.e., the total number and the main comorbidity, as well as information about vaccination status against COVID-19 and possible hospital admission. The data were entered into a database created by the Institute for Public Health of Vojvodina, Novi Sad.

The time period of seven pandemic waves and the distribution of SARS-CoV-2 variants in Serbia during the entire period under observation are described elsewhere [23]. Briefly, the first pandemic wave (6 March–1 June 2020) had a predominance of the 19A variant; the second wave (2 June–6 October 2020) had variant 20A; the third, the 20E (EU1) variant (7 October 2020–31 January 2021); fourth, the 20I (Alpha, V1) variant (1 February–23 July 2021); in the fifth, the Delta variant was dominant, 21J (24 July–31 December 2021); the sixth was dominated by 21K, 21L (Omicron) variant (1 January–30 June 2022), and the seventh pandemic wave (1 July 2022–31 December 2022) had dominant variant 22B (Omicron) [24].

According to the clinical presentation of COVID-19, cases have been classified into four categories: (1) asymptomatic cases, if there were no signs or symptoms of the disease at the time of testing and/or epidemiological interview; (2) mild, if any of the respiratory, digestive, or general infectious symptoms were present in the laboratory-confirmed case, and there were no clinical or radiological signs of pneumonia present; (3) severe, if in addition to the presence of symptoms and signs of COVID-19, there were clinical and radiological signs of confirmed pneumonia; and (4) critical case, which refers to the present COVID-19 pneumonia requiring intensive care treatment with intubation and/or invasive mechanical ventilation [25]. On the other hand, COVID-19 reinfection was defined as a laboratory-confirmed SARS-CoV-2 infection by RT-PCR or Ag-RDT in a person after ≥90 days from the confirmed primary infection, regardless of the presence of symptoms and signs at the time of testing [26].

The vaccination status was predicated upon four available vaccines: namely Pfizer-BioNTech BNT162b2 (Comirnaty^®^), Sinopharm BBIBP-CorV (Vero Cell^®^), Gam-COVID-Vac (Sputnik V^®^) and Oxford/AstraZeneca ChAdOk1-S/nCoV-222 (Vaxzevria^®^) [27]. Patients were categorized as non-vaccinated if they had not received any dose, as having received one dose, and as having received two or three doses, according to their vaccination status at the time of confirmed SARS-CoV-2 (re)infection. Specifically, individuals were designated as non-vaccinated if they had not received any dose or had received the first dose (or the first of two doses regimen) ≤ within a window of 14 days prior to infection or if the time span between the first and second dose was >6 months and ≤14 days since the second dose at the time of infection. Vaccination with one dose means receiving a single dose of the vaccine with a lapse of more than 14 days since its administration, the vaccination scheme has not yet been completed, or the second dose has been administered ≤14 days or >6 months after the first dose. Vaccination with two doses was considered when two doses of the vaccine were received, with the last dose being administered >14 days and ≤6 months prior to the infection. Finally, the reception of the third dose (commonly referred to as a booster dose) was considered when a patient had this additional dose >14 days before the onset of the infection and when the time between the second and the first dose did not exceed 6 months. It is noteworthy that all patients infected with SARS-CoV-2 infection before January 2021 (when COVID-19 vaccines became widely available in Serbia) were considered non-vaccinated at the time of infection.

### 2.4. Statistical Analyses

We used descriptive statistics to analyze the socio-demographic, epidemiological and clinical features of individuals testing positive for the SARS-CoV-2. Age-specific incidence rates of COVID-19 for the North Bačka district were calculated using the number of cases in the district across the defined age categories as the numerator and the population of the district under surveillance according to the census for the specific age category [21], as the denominator, and multiplied per 10,000 or 100,000 inhabitants. For the purpose of statistical analyses, patients were further classified into the following groups: “working-age group” (18–64 years) and “retirement-age group” (≥65 years). To assess differences in distribution between the groups, we used the chi-squared (χ^2^) or Fisher’s exact test (where appropriate) or ANOVA test for categorical, and the *t*-test for continuous and discrete variables.

Additionally, we used the multivariable Cox proportional hazard regression model to explore the risk of SARS-CoV-2 reinfection with respect to the selected explanatory factors. The results are expressed as an adjusted hazard ratio (aHR) with the corresponding 95% confidence interval (95% CI). All statistical analyses were conducted using statistical software Stata v.16 (STATA StataCorp, College Station, TX, USA) and results with the *p*-value < 0.05 were considered statistically significant.

### 2.5. Ethical Considerations

In accordance with the applicable laws and regulations in Serbia, there is no requirement for approval from the Ethics Committee for retrospective analysis of anonymous data. The authors were not involved in the treatment of the included patients and did not have access to information that could identify individual participants during and after data collection. The data were accessed for research purposes on 20 January 2023.

## 3. Results

**Figure 1:**
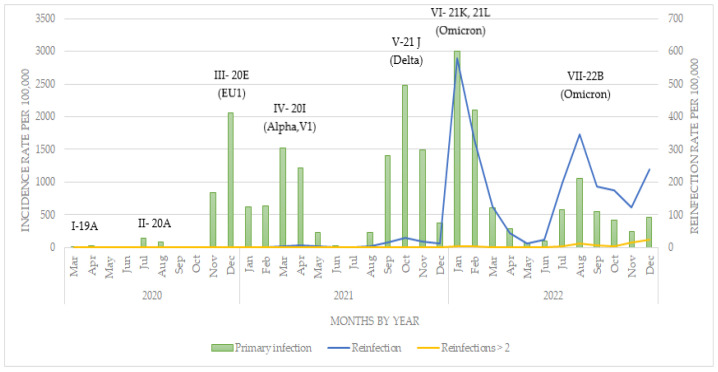
Incidence rate of SARS-CoV-2 primary infections and reinfections in the North Bačka district, Serbia, 2020–2022.

During the observed period, a total of 38,685 SARS-CoV-2 primary infections and 4067 reinfections were reported in the North Bačka district across seven pandemic waves. The incidence rate of COVID-19 among the population of the North Bačka district, categorized by the month of disease registration, is shown in Figure 1. Low incidence rates were observed during the first two pandemic waves, while the highest incidence rates were recorded in October 2021 (2482.37/100,000 inhabitants) and January 2022 (2994.45/100,000 inhabitants), with the predominance of the Delta and Omicron variants, respectively. As of the onset of 2022 and the shift in predominance to the Omicron variant, an incidence rate of 579.41 reinfections per 100,000 inhabitants was reported. This represents the highest rate of reinfections recorded throughout the entire period under surveillance (2020–2022).

Socio-demographic, epidemiological, and clinical characteristics of patients with confirmed primary SARS-CoV-2 infection are shown in Table 1. Over the observed period, the largest number of cases was recorded in 2021 (*n* = 17,905), followed by 15,114 cases in 2022 and 5666 in 2020. The mean age of all patients at the time of infection was 48.15 (SD = 18.92) years, ranging from 47.05 (SD = 18.76) in 2021, 47.89 (SD = 16.29) in 2020 to 49.55 (SD = 19.90) years in 2022 (*p* < 0.001). A majority of patients were women (54.1%), with the highest percentage notified in 2022 (55.68%) (*p* < 0.001). Approximately 83% were residents of Subotica, the most populous municipality of the district, and around 4% were healthcare workers (HCWs) by occupation. Throughout the observed period, nearly 94% of the total laboratory-confirmed cases were detected using the Ag-RDT. Clinical presentation of COVID-19 varied throughout the surveillance period, with a predominance of the mild form (81.63% in 2020, 87.50% in 2021, and 95.02% in 2022) (*p* < 0.001). The proportion of patients with comorbidities increased over the surveillance period, rising from 26.95% in 2020 to 30.27% in 2021 and further to 38.90% in 2022 (*p* < 0.001). Notably, the prevalent comorbidities were hypertension (49.17%) followed by diabetes (9.85%) and cardiovascular disease (8.67%).

Following the introduction of the COVID-19 vaccine at the beginning of 2021, the majority of individuals testing positive for SARS-CoV-2 in that year were unvaccinated (84.54%) at the time of infection, while around 12% of participants had received two doses. In 2022, there were 62.98% of unvaccinated cases, followed by those who had received three doses (33.03%) and two doses (3.54%). Among the total of 8363 patients who received at least one dose of the vaccine, the most widely administered vaccine (two doses in primary series) in this cohort of SARS-CoV-2 positive participants was BBIBP-CorV (67.45%), followed by BNT162b2 (19.81%), Gam-COVID-Vac (9.31%), and ChAdOx1 nCoV-19 (3.42%) vaccine.

**Figure 2:**
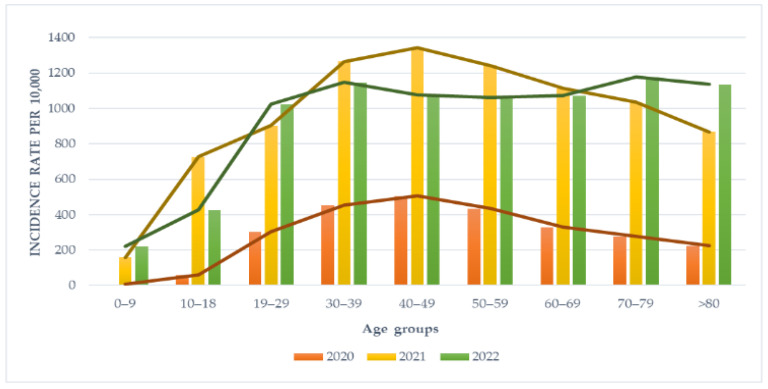
Age-specific incidence rates of SARS-CoV-2 primary infections in the North Bačka district, Serbia, 2020–2022.

The age-specific incidence rates of SARS-CoV-2 primary infections are shown in Figure 2. Notably, the highest incidence rates were recorded in 2021 across almost all age groups, with peaks in the 40–49 age category (1345.32 per 10,000 inhabitants), 30–39 age category (1265.95 per 10,000 inhabitants), and 50–59 age category (1242.38 per 10,000 inhabitants). Similarly, most infections occurred within age categories ranging 30–39 to 60–69 in 2020. Conversely, in 2022, a shift towards the older population (age categories ≥ 60 years old) was observed, with the highest incidence rates recorded in the 70–79 age group (1178.03 per 10,000 inhabitants) and ≥80 age group (1135.42 per 10,000 inhabitants). Although the pediatric population (<10 years old) became the least affected during the entire surveillance period, an increase in incidence was observed for the years 2021 and 2022 compared to 2020.

Upon an investigation into the population ≥ 18 y years old (*n* = 36,480), with a particular focus on the differences between the working-age population and those of the retirement age, it was observed that there was no statistically significant difference regarding the gender of participants (*p* = 0.333) (Table 2). A higher percentage of severe (17.26% vs. 4%) and critical (0.68% vs. 0.1%) cases was evident in the retirement-age category compared to the working age group, respectively (*p* < 0.001). A significantly higher number of patients from the retirement-age category had at least one comorbidity (70.68%) compared to the working-age population (23.71%) (*p* < 0.001). The reported main type of comorbidity was hypertension in both groups (54.72% and 44.68%, respectively). A higher percentage of unvaccinated participants was reported in the working-age population (82.62%) in comparison to the other category (59.53%) (*p* < 0.001). The most widely administered vaccine in both groups was the BBIBP-CorV (58.47% in the working-age category and 80.02% in retirement-age category). Among the total cases aged ≥18 years, there were 10.64% (3883 out of 36,480) patients with one or more reinfections. While a higher number of reinfections was recorded among the working-age population in comparison to the retirement age category, the proportions of individuals with one, two or three reinfections were not significantly different (*p* = 0.152).

**Figure 3:**
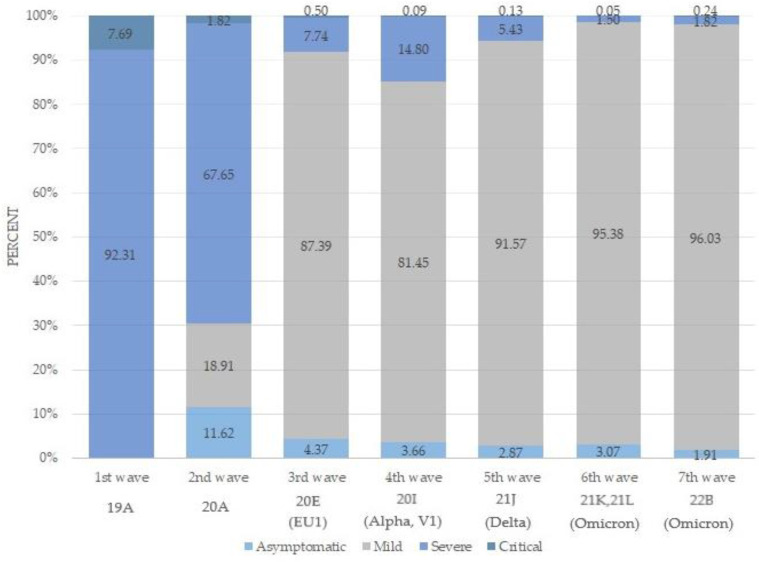
Clinical forms of COVID-19 by pandemic waves in the North Bačka, Serbia, 2020–2022.

The clinical forms of COVID-19 cases notified during seven consecutive pandemic waves are shown in Figure 3. In the first wave, characterized by the circulation of the original SARS-CoV-2 Wuhan variant, only severe (92.31%) and critical (7.69%) forms of the disease were reported. Subsequent waves brought an increase in the percentage of mild cases, starting from 18.91% in the second wave to over 91% in the fifth wave, when the Delta variant became dominant. The severity of cases started to plummet with the arrival of the Omicron. Nevertheless, it is important to note that during the period of Delta variant transmission, severe disease was still a possibility partially because not all individuals had received two vaccine doses. This trend continued with the rise of the Omicron variant during the sixth (95.38%) and seventh (96.03%) waves. Conversely, the percentage of asymptomatic cases, apart from the second wave (11.62%), remained consistently low, accounting for less than 5% of the notified cases throughout the entire investigated period.

There was a total of 3961 patients with one reinfection, and 106 patients faced two reinfections, with the majority (3910 out of 4067, 96.14%) being notified in 2022. Patients with one reinfection were significantly older in comparison to those who had two infections (mean age = 47.32, SD = 15.38 vs. mean age = 44.62, SD = 12.94, respectively) (*p* = 0.045). The occurrence of the second reinfection was recorded only in 2022 (*n* = 106, 100%). In both groups, the majority of reinfections were with mild clinical presentation, accounting for 97.78% in the group with one reinfection and 97.17% in the group with two reinfections (*p* = 0.586). A higher percentage of individuals with comorbidities was observed in the group with two reinfections (42.45%) compared to the group with one reinfection (37.41%) (*p* = 0.516). Among those with one reinfection, 26.29% had been vaccinated, while 23.58% of those with two reinfections had been vaccinated. The majority had received three vaccine doses at the time of reinfection (21.21% and 22.64%, respectively) (*p* = 0.294), and the most frequently administered vaccine was BBIBP-CorV in both groups (65.03% in the one-reinfection group and 64% in the two-reinfection group) (*p* = 0.464). Detailed characteristics of patients who had reinfection(s) are given in Table S1.

Table 3 presents results from multivariable Cox regression analysis exploring the risk of reinfection in our population during the surveillance period. The risk of reinfection was lower for male patients in comparison to females (HR = 0.79, 95% CI: 0.74–0.85) and for all occupation categories in comparison to HCW (service provider HR = 0.57, 95% CI: 0.48–0.67; retirement HR = 0.44, 95% CI: 0.38–0.51, other HR = 0.58, 95% CI: 0.51–0.66). Older age categories demonstrated higher risk with respect to the youngest category, ranging from the smallest risk in 10–18 years old (HR = 2.36, 95% CI: 1.14–4.88) to the largest in the 30–39 years old (HR = 5.76; 95% CI: 2.87–11.56). When exploring the effect of severity of the primary SARS-CoV-2 infection on the risk of reinfection, it was noticed that those with a mild clinical presentation had significantly higher risk in comparison to those asymptomatic (HR = 1.25, 95% CI: 1.03–1.52). On the other hand, those vaccinated with two doses (complete regiment) at the time of primary COVID-19 had a lower risk (HR = 0.72, 95% CI: 0.62–0.84) in comparison with the unvaccinated.

Finally, when investigating the effect of pandemic wave when primary COVID-19 occurred, those infected during the second wave (predominance of the 20A.EU1 variant) had lower risk (HR = 0.67, 95% CI: 0.49–0.91) while those infected during the following waves had higher risk, from HR = 1.15, 95% CI: 1.04–1.28 in the third wave up to HR = 1.41 (95% CI: 1.25–1.60) in the sixth wave. When further adjusting the analyses for the effect of other confounders (age category, sex, pandemic wave, severity of primary COVID-19, and the time passed from the last dose of vaccine to reinfection) across the three explored models, the results remained stable with some modest change in the effects, as presented in Table 3.

## 4. Discussion

In this cross-sectional study, we analyzed the epidemiological and clinical characteristics of laboratory-confirmed cases of COVID-19 in the northern region of Vojvodina, Serbia. To the best of our knowledge, this is the first study that represents the comprehensive investigations of COVID-19 cases in the North Bačka district during seven consecutive pandemic waves. Due to the importance of the district in terms of economy and travel, the implementation of epidemiological surveillance for SARS-CoV-2 has yielded valuable data. A continuous analysis of epidemiological waves over time, coupled with an analysis of dominant variants as well as vaccination status, has never been conducted for this district.

At the end of February 2020, Italy became the center of the COVID-19 pandemic in Europe [28]. Not long after, the first confirmed cases of COVID-19 in the region were reported in the Republic of Croatia—26 February 2020, the Federation of Bosnia and Herzegovina—29 February 2020, the Republic of Slovenia—4 March 2020, Hungary on 4 March 2020, and the Republic of Serbia—6 March 2020 [5,28,29]. In response, the Republic of Serbia declared a state of emergency, leading to the implementation of the following measures: border closures, movement restrictions, the closure of educational institutions, and a recommendation for residents over 65 years of age to remain indoors. The state of emergency was lifted on 6 May 2020. Serbia, in contrast to its regional counterparts, reopened the borders and eased the measures, resulting in the emergence of a second epidemiological wave [5,28]. By the time the third wave occurred, the community was already experiencing widespread transmission of SARS-CoV-2 [5]. The beginning of our study coincides with the registration of the first case of COVID-19 in the country, which followed after the neighboring countries had already notified their first cases. The implementation of the measures certainly contributed to a smaller number of patients at the beginning of the epidemic; however, their relaxation subsequently led to a surge in reported numbers, which was evident in the second and third epidemiological waves.

Our study reveals that, cumulatively, during the period of surveillance, women were more infected than men, which was also detected on an annual basis and in the cases of reinfection. Pijls et al., in their meta-analysis, found that men were at a higher risk of contracting SARS-CoV-2 infection [30], which was also confirmed by the study in a highly vaccinated population of HCWs during the first year of the vaccination campaign (before Omicron) [13]. However, other studies have demonstrated a higher percentage of female patients in the population of COVID-19 patients [14,15,31,32,33,34]. Globally, a large proportion of the health and social care workforce is represented by women, exposing them to a higher risk for morbidity, both at work and at home, when caring for sick family members [35,36].

Our research indicates that comorbidities were more frequent in the retirement-age group compared to the working-age population, which reflects a usual finding in this age group. This disparity, together with immunosenescence, a phenomenon of declining immunity with age, can additionally result in lower immunogenicity and effectiveness of vaccines in this group [37]. As a consequence, those in this group are at increased risk of developing severe forms of COVID-19 and poorer outcomes [11]. Notably, in Vojvodina, a significant presence of hypertension is observed in the population older than 65 years, especially among those older than 74 years, where the share of hypertension was higher in those not immunized against COVID-19 [38]. In a recent meta-analysis, which included 28 studies on 6276 individuals, Honardoost et al. revealed that 41.1% of respondents had associated comorbidities, of which hypertension accounted for 20.9%. The study indicated that the existence of previous comorbidities makes patients with COVID-19 more susceptible to developing severe form [39]. Additionally, it was observed that the risk for breakthrough infection was higher in patients with diabetes mellitus and BMI > 25 [13].

Our findings showed that the working-age population was more affected during the first five waves of the epidemic in terms of primary infections and re-infections. This can be attributed to the stringent measures implemented during the state of emergency in the Republic of Serbia, which related to the elderly population and included the recommendation for people ≥ 65 years old to stay at home [5]. Furthermore, being at a higher risk for developing more serious forms of COVID-19, the older population was expected to adhere to these measures and wear masks more often than the younger population [40].

In contrast, during the last two epidemic waves, a shift of the age limit towards the older was observed. A possible explanation could be that anti-spike IgG to SARS-CoV-2 virus decreases over time, particularly considering the seropositivity to SARS-CoV-2 varied depending on the received vaccine. Some studies reported seropositivity rates below 40% for BBIBP-CorV, which was the most widely administered in our study of elderly patients [41]. Due to the exacerbating of pre-existing comorbidities, some symptoms of COVID-19 may have been manifested earlier [38]. Social factors may have also contributed to the higher circulation of viruses due to the relaxation of non-pharmaceutical measures [42].

The appearance of new variants of SARS-CoV-2 (Alpha, Delta, and Omicron) also affected the dynamics for primary infections and reinfections, resulting in a fluctuation in the incidence rate, which reached its peak during the corresponding periods. While the initial cases of reinfections were notified at the end of the first year of surveillance, the occurrence of the second reinfection was particularly prominent in 2022, coinciding with their maximum number on two occasions. These reinfections were more frequent in the middle-aged population and females, and they were distinguished by a mild clinical form. The risk of transmission appeared to increase over time, varying among different variants (original variant > 45–71% for Alpha > 40–60% for Delta), whereby Omicron is ~3.2 times higher compared with the Delta strain, but it is less virulent [15,43,44,45]. Compared with other variants, Omicron certainly has more mutations, and these mutations may cause reduced antibody binding affinity and allow for enhanced immune escape [46,47]. Additionally, to assist in classical epidemiological surveillance, monitoring of wastewater has proven useful since the presence of the virus can be detected in stool for those symptomatic and asymptomatic cases, thus including unreported ones [48].

A meta-analysis has indicated that protection acquired during prior infection was high for the original, Alpha, Beta and Delta variants, while lower values were observed for the Omicron BA.1 variant [49]. However, during the pre-Omicron period, in the case of primary infection, the risk for reinfection was lower in the population < 60, while in case the Omicron infection was the primary infection, the population aged 30–50 years faced a higher risk [15]. The vaccine effectiveness of the booster dose decreased from 90% before the appearance of Omicron to 63% after its emergence [50].

Among individuals aged ≥18 years old, the share of reinfections in our study was 10.64%, while for the entire population of Vojvodina, Medić et al. reported an overall incidence rate of 5.49% population with documented pediatric reinfections of 3.2% [23,26]. Furthermore, Medić and his colleagues conducted a study on reinfections recorded in Vojvodina during the period 2020–2022, where they pointed out that the largest number of notified reinfections occurred during January 2022. Prior to this period, sporadic occurrences of reinfection were noted. However, with the emergence of Omicron, the risk of reinfection began to increase, and the risk of reinfection was higher in women and middle-aged people [26]. These findings are in concordance with our research.

The majority of patients in our study were unvaccinated at the time of the COVID-19 diagnosis. The first approved vaccine in Serbia, on 23 December 2020, was Pfizer-BioNTech BNT162b2, marking the beginning of the national vaccination campaign. Subsequently, additional vaccines became available in the following weeks, in particular, Sinopharm BBIBP-CorV, followed by Gam-COVID-Vac, and the Oxford/AstraZeneca ChAdOk1-S/nCoV-19 AZD1222 [25]. Moderna mRNA-1273 (Spikevax^®^) vaccine was introduced later in November 2021 [40]. At the moment of (re)infection in our study, among those vaccinated, the most frequent was the BBIBP-CorV vaccine. This may be attributed to the potentially lower effectiveness of this vaccine. After administration of two doses of the BBIBP-CorV vaccine 21 days apart, its effectiveness was previously estimated at 73.78% [51]. Based on studies done in Vojvodina, early effectiveness in the prevention of COVID-19 in the elderly after two applied doses of BBIBP-CorV was 86.9%, while the prevention of the most serious forms was 90.5% [27]. Additionally, Petrović and his colleagues, in their longitudinal study, monitored seropositivity after 28 days and six months after the second dose of different vaccines and demonstrated a significant decline in the antibody levels, most prominent in individuals vaccinated with BBIBP-CorV vaccine, which could indicate that the BBIBP-CorV show weaker protection over time [41]. Levin et al. monitored the humoral response in those vaccinated with the BNT162b2 vaccine over six months and observed lower IgG titer and neutralizing antibody levels among the population older than 65 years and those with multiple comorbidities (two or more) [52]. Furthermore, different levels of antibodies were observed among individuals with specific comorbidities, such as patients on dialysis and other chronic kidney diseases [53]. Additionally, lower levels of IgG antibodies were noted among men [52,54].

The proportion of different clinical forms of COVID-19 changed through pandemic waves, reflecting several key factors. These include the characteristics of the dominant viral strain, gained immunity in the population, as well as the increased availability of diagnostic tools and implementation of anti-epidemic measures at the time. During our study period, the majority of patients had a mild clinical form, except throughout the first wave, when predominantly severe and critical forms of the disease were reported. The registration of those forms of the disease during the first wave can be attributed to the fact that only hospitalized patients were laboratory-tested for COVID-19 during the initial wave. Such selective testing in Serbia was conducted under “Algorithm 5”, at the beginning of the epidemic [55]. In alignment with these findings, as previously described in the SARS-CoV-2 seroprevalence study among asymptomatic subjects, there were approximately 76,000 individuals in Vojvodina with SARS-CoV-2 antibodies during the complete lockdown period (which coincided with the first wave), yet only 875 COVID-19 cases were officially notified [56]. As the availability of Ag-RDT tests increased in Serbia, the testing algorithm was expanded so that other clinical forms became more present throughout the subsequent waves, depending on the dominant strain that was circulating. In a subsequent study that focused on health workers in state health centers (6936 participants out of 27,738 of all employees in Vojvodina), comparing the clinical manifestations they had the last time they were SARS-CoV-2 positive with their vaccination status, it was found that the highest seroprevalence of SARS-CoV-2 antibodies had asymptomatic (64.36%), mild (45.17%), severe (38.39%), and critical (45.45%) forms that received three doses of the vaccine [57].

Lin et al. [43] demonstrated that the risk for hospitalization and admission to ICU differs depending on the SARS-CoV-2 variant—in terms of hospitalization, the Beta variant had the highest risk with 2.16 (95% CI: 1.19–3.14), while for admission to the ICU, the Delta strain demonstrated the highest risk (3.35, 95% CI: 2.5–4.2) [43]. A comparative analysis between the Omicron and Delta variants, based on the results of 42 studies covering 6,174,807 patients, conducted by Relan et al., concluded that patients infected with Omicron had a 56% lower risk of hospitalization, a 54% lower risk of ICU admission, and a 61% lower risk of death [58].

Several limitations should be taken into account when interpreting our results. Firstly, at the very beginning of the epidemic, testing capacities were limited, potentially resulting in an underestimation of the actual number of COVID-19 cases. However, the limitation in testing capacities was later successfully overcome due to the greater availability of the Ag-RDT. Secondly, there is a possibility that some data in the epidemiological questionnaire, especially comorbidities, may not have been consistently recorded despite the previously organized training that the healthcare workers underwent. Thirdly, immunocompromised conditions are not separated in the framework of leading comorbidities but are rather included under other conditions. Insisting on data on comorbidities, as well as emphasizing the existence of immunocompromised conditions, would provide a better insight into the outcome. Fourthly, there is a possibility that certain persons were vaccinated abroad, which cannot be recognized because the vaccination data programs are not linked and integrated. Fifthly, with regard to the administration of the third dose of the vaccine against COVID-19, the patient was allowed to choose the preferred vaccine, therefore potentially leading to different effectiveness depending on the combination of the primary series and the booster. Consequently, monitoring the vaccination status of those vaccinated in other countries and in the district, based on the primary series and booster doses of the vaccine, would provide valuable data for further research on vaccine effectiveness.

## 5. Conclusions

Our findings offer insight into the general epidemiological and clinical dynamics of SARS-CoV-2 infections in the North Bačka district during the first three years of the COVID-19 pandemic. The imposition of strict measures at the beginning of the epidemic of COVID-19 in the Republic of Serbia reduced the number of confirmed cases in the North Bačka district during the first epidemic wave. The mitigation of epidemiological measures and the emergence of new variants of SARS-CoV-2 resulted in an increase in the number of cases and the start of new waves of the epidemic, both at the national and the regional levels, including North Bačka.

It was demonstrated that the presence of the Alpha, Delta and Omicron SARS-CoV-2 variants resulted in the high incidence rate of COVID-19, with females being more frequently infected. Our results indicate that the highest number of patients was recorded in the working-age population. Notably, in the period when Omicron was dominant, the infection slowly moved towards older age groups. Furthermore, it was noticed that the disease predominately manifested in a mild clinical form, except during the first two waves. The majority of patients were unvaccinated, but the proportion of unvaccinated decreased during the surveillance period. Statistically, a significantly higher number of unvaccinated was among the working population compared to the retired, while those vaccinated (two doses of vaccine in the primary series) in the highest number of cases received the BBIBP-CorV vaccine. The majority of reinfections were notified during 2022, whereby patients with a second reinfection more often had at least one comorbidity. Encouragingly, both the first and the second reinfection cases predominantly displayed mild clinical form. Managing this pandemic provided valuable lessons that could serve as a useful resource in shaping future strategies for addressing similar threats within the region.

## Figures and Tables

**Table 1:** General characteristics of patients based on the year of COVID-19 primary infection.

	Total (*n* = 38,685)				
		2020 (*n* = 5666)	2021 (*n* = 17,905)	2022 (*n* = 15,114)	*p*-Value ¹
**Sex, *n* (%)**					
Male	17,757 (45.9)	2827 (49.89)	8231 (45.97)	6699 (44.32)	**<0.001**
Female	20,928 (54.1)	2839 (50.11)	9674 (54.03)	8415 (55.68)	
**Age at infection, years**, mean (SD)	48.15 (18.92)	47.89 (16.29)	47.05 (18.76)	49.55 (19.90)	**<0.001**
**Municipality, *n* (%)**					
Bačka Topola	3900 (10.08)	610 (10.77)	1614 (9.01)	1676 (11.09)	**<0.001**
Mali Iđoš	2621 (6.78)	395 (6.97)	1269 (7.09)	957 (6.33)	
Subotica	32,164 (83.14)	4661 (82.26)	15,022 (83.9)	12,481 (82.58)	
**Occupation, *n* (%)**					
Service provider	2193 (5.67)	669 (11.81)	1127 (6.29)	397 (2.63)	**<0.001**
Healthcare worker	1516 (3.92)	467 (8.24)	539 (3.01)	510 (3.37)	
Retirement	9791 (25.31)	1132 (19.98)	4154 (23.2)	4505 (29.81)	
Other	25,185 (65.1)	3398 (59.97)	12,085 (67.5)	9702 (64.19)	
**Type of COVID-19 test, *n* (%)**					
RT-PCR	2529 (6.54)	1329 (23.46)	736 (4.11)	464 (3.07)	**<0.001**
Ag-RDT	36,156 (93.46)	4337 (76.54)	17,169 (95.89)	14,650 (96.93)	
**Clinical presentation of COVID-19, *n* (%)**					
Asymptomatic	1290 (3.33)	308 (5.44)	543 (3.03)	439 (2.90)	**<0.001**
Mild	34,652 (89.57)	4625 (81.63)	15,666 (87.50)	14,361 (95.02)	
Severe	2655 (6.86)	702 (12.39)	1662 (9.28)	291 (1.93)	
Critical	88 (0.23)	31 (0.55)	34 (0.19)	23 (0.15)	
**Comorbidity number, *n* (%)**					
None	25,859 (66.85)	4139 (73.05)	12,485 (69.73)	9235 (61.1)	**<0.001**
One	9622 (24.87)	1214 (21.43)	4191 (23.41)	4217 (27.9)	
Two	2503 (6.47)	260 (4.59)	986 (5.51)	1257 (8.32)	
Three or more	701 (1.81)	53 (0.94)	243 (1.36)	405 (2.68)	
**Type of main comorbidity, *n* (%)**					
Obesity	557 (4.34)	52 (3.41)	299 (5.52)	206 (3.5)	**<0.001**
Diabetes	1263 (9.85)	155(10.15)	570 (10.52)	538 (9.15)	
Hypertension	6307 (49.17)	771 (50.49)	2652 (48.93)	2884 (49.06)	
Malignant disease	313 (2.44)	32 (2.10)	134 (2.47)	147 (2.50)	
Cardiovascular disease	1112 (8.67)	181 (11.85)	420 (7.75)	511 (8.69)	
Chronic lung disease	918 (7.16)	116 (7.60)	399 (7.36)	403 (6.85)	
Other chronic disease or condition	2356 (18.37)	220 (14.41)	946 (17.45)	1190 (20.24)	
**Vaccination status at the time of infection, *n* (%)**					
Unvaccinated	30,322 (78.38)	5666 (100)	15,137 (84.54)	9519 (62.98)	**<0.001**
One dose	348 (0.9)	0	280 (1.56)	68 (0.45)	
Two doses	2700 (6.98)	0	2165 (12.09)	535 (3.54)	
Three doses (booster)	5315 (13.74)	0	323 (1.80)	4992 (33.03)	
**Type of vaccine in the primary-vaccination (two doses of vaccine), *n* (%)**	8363 (100)	NA	2768 (100)	5595 (100)	NA
ChAdOx1 nCoV-19	286 (3.42)	NA	112 (4.05)	174 (3.11)	**<0.001**
BNT162b2	1657 (19.81)	NA	458 (16.55)	1199 (21.43)	
BBIBP-CorV	5641 (67.45)	NA	1975 (71.35)	3666 (65.52)	
Gam-COVID-Vac	779 (9.31)	NA	223 (8.06)	556 (9.94)	

^1^ Indicators of significance between the groups using Pearson’s chi-squared test and Fisher’s exact test (where appropriate) for categorical variables and Kruskal–Wallis equality-of-populations rank test for continuous variables. Significance levels are given in bold for *p* < 0.05. NA—not applicable. *n*—number of participants.

**Table 2:** General characteristics of the COVID-19 cases by work/retirement status, 2020–2022.

	Working-Age (18–64 yrs Old) *n* = 27,785	Retirement-Age (≥65 yrs Old) *n* = 8695	*p*-Value ^1^
**Sex, *n* (%)**			
Male	12,707 (45.73)	3925 (45.14)	0.333
Female	15,078 (54.27)	4770 (54.86)	
**Age at infection, years**, mean (SD)	43.20 (12.46)	73.25 (6.49)	NA
**Municipality, *n* (%)**			
Bačka Topola	2647 (9.53)	1043 (12.00)	**<0.001**
Mali Iđoš	1765 (6.35)	608 (6.99)	
Subotica	23,373 (84.12)	7044 (81.01)	
**Type of COVID-19 test, *n* (%)**			
RT-PCR	1768 (6.36)	698 (8.03)	**<0.001**
Ag-RDT	26,017 (93.64)	7997 (91.97)	
**Clinical presentation of COVID-19, *n* (%)**			
Asymptomatic	930 (3.35)	293 (3.37)	**<0.001**
Mild	25,715 (92.55)	6842 (78.69)	
Severe	1111 (4.00)	1501 (17.26)	
Critical	29 (0.10)	59 (0.68)	
**Comorbidity number, *n* (%)**			
None	21,198 (76.29)	2549 (29.32)	**<0.001**
One	5394 (19.41)	4138 (47.59)	
Two	977 (3.52)	1523 (17.52)	
Three or more	216 (0.78)	485 (5.58)	
**Type of main comorbidity ^#^** **, *n* (%)**			
Obesity	417 (6.33)	103 (1.68)	**<0.001**
Diabetes	590 (8.96)	671 (10.92)	
Hypertension	2943 (44.68)	3363 (54.72)	
Malignant disease	143 (2.17)	167 (2.72)	
Cardiovascular disease	401 (6.09)	709 (11.54)	
Chronic lung disease	621 (9.43)	286 (4.65)	
Other chronic disease or condition	1472 (22.35)	847 (13.78)	
**Vaccination status at the time of infection, *n* (%)**			
Unvaccinated	22,955 (82.62)	5176 (59.53)	**<0.001**
One dose	216 (0.78)	130 (1.50)	
Two doses	2026 (7.29)	662 (7.61)	
Three doses (booster)	2588 (9.31)	2727 (31.36)	
**Type of vaccine in the primary-vaccination (two doses of vaccine) ** **(*n* = 8349)**	**4830 (100)**	**3519 (100)**	**NA**
ChAdOx1 nCoV-19	174 (3.60)	112 (3.18)	**<0.001**
BNT162b2	1288 (26.67)	356 (10.12)	
BBIBP-CorV	2824 (58.47)	2816 (80.02)	
Gam-COVID-Vac	544 (11.26)	235 (6.68)	
**Reinfection, *n* (%)**			
One reinfection	3197 (97.11)	582 (98.48)	0.152
Two reinfections	93 (2.83)	9 (1.52)	
Three reinfections	2 (0.06)	0	

^1^ Indicators of significance between groups using Pearson’s chi-squared test and Fisher’s exact test (where appropriate). Significance levels are given in bold for *p* < 0.05. ^#^ The main comorbidity is presented for those having multiple comorbidities. NA—not applicable. *n*—number of participants.

**Table 3:** Cox proportional regression analysis for the risk of SARS-CoV-2 reinfection.

		Model 1	Model 2	Model 3
	HR (95% CI)	aHR (95% CI)	aHR (95% CI)	aHR (95% CI)
**Sex**				
Male	**0.79 (0.74–0.85)**	-	-	-
Female	ref.	-	-	-
**Age category**				
0–9	ref.	-	-	-
10–18	**2.36 (1.14–4.88)**	-	-	-
19–29	**4.48 (2.22–9.03)**	-	-	-
30–39	**5.76 (2.87–11.56)**	-	-	-
40–49	**5.45 (2.72–10.94)**	-	-	-
50–59	**5.48 (2.73–11.01)**	-	-	-
>60	**4.04 (2.01–8.11)**	-	-	-
**Occupation**				
Service provider	**0.57 (0.48–0.67)**	**0.59 (0.50–0.70)**	**0.59 (0.50–0.70)**	**1.43 (1.07–1.91)**
Healthcare worker	ref.	ref.	ref.	ref.
Retirement	**0.44 (0.38–0.51)**	**0.38 (0.32–0.44)**	**0.37 (0.32–0.43)**	1.22 (0.94–1.58)
Other	**0.58 (0.51–0.66)**	**0.63 (0.55–0.72)**	**0.62 (0.54–0.70)**	**1.28 (1.04–1.58)**
**Clinical presentation of primary infection**				
Asymptomatic	ref.	ref.	ref.	-
Mild	**1.25 (1.03–1.52)**	**1.26 (1.04–1.54)**	**1.26 (1.04–1.53)**	-
Severe	0.91 (0.71–1.15)	0.92 (0.73–1.17)	0.93 (0.73–1.18)	-
Critical	0.78 (0.11–5.64)	0.77 (0.11–5.54)	0.78 (0.11–5.61)	-
**Comorbidity number**				
None	ref.	ref.	ref.	ref.
One	1.06 (0.98–1.15)	1.05 (0.96–1.14)	1.04 (0.96–1.14)	1.00 (0.86–1.17)
Two	1.11 (0.95–1.29)	1.09 (0.93–1.28)	1.09 (0.92–1.28)	0.99 (0.78–1.28)
Three or more	1.16 (0.85–1.58)	1.14 (0.83–1.56)	1.13 (0.83–1.55)	1.96 (1.12–3.42)
**Time passed from last dose of vaccine to reinfection (in weeks)**	**0.95 (0.95–0.96)**	**0.95 (0.95–0.96)**	**0.93 (0.92–0.93)**	-
**Vaccine at the time of primary infection**				
Unvaccinated	ref.	ref.	ref.	ref.
One dose	**1.47 (1.11–1.95)**	**1.47 (1.10–1.96)**	**1.45 (1.09–1.92)**	**2.01 (1.31–3.09)**
Two doses	**0.72 (0.62–0.84)**	**0.71 (0.61–0.84)**	**0.70 (0.60–0.82)**	**3.40 (2.48–4.66)**
Three doses (booster)	**1.36 (1.16–1.59)**	**1.33 (1.14–1.56)**	**1.28 (1.08–1.52)**	NA
**Type of vaccine in the primo-vaccination (two doses of vaccine)**				
ChAdOx1 nCoV-19	0.64 (0.32–1.30)	0.67 (0.33–1.36)	0.67 (0.33–1.36)	1.45 (0.27–7.86)
BNT162b2	0.97 (0.75–1.27)	0.87 (0.66–1.15)	0.85 (0.64–1.11)	2.30 (1.06–4.98)
BBIBP-CorV	ref.	ref.	ref.	ref.
Gam-COVID-Vac	1.05 (0.74–1.47)	1.02 (0.72–1.43)	0.99 (0.71–1.41)	1.00 (0.48–2.11)
**Pandemic wave at the time of primary infection**				
First	1.30 (0.80–2.11)	1.33 (0.82–2.15)	-	**0.01 (0.01–0.01)**
Second	**0.67 (0.49–0.91)**	**0.68 (0.50–0.92)**	-	**0.01 (0.01–0.02)**
Third	**1.15 (1.04–1.28)**	**1.16 (1.05–1.28)**	-	**0.06 (0.04–0.08)**
Fourth	**1.36 (1.23–1.50)**	**1.35 (1.22–1.49)**	-	**0.17 (0.12–0.24)**
Fifth	ref.	ref.	-	ref.
Sixth	**1.41 (1.25–1.60)**	**1.41 (1.25–1.59)**	-	**7.01 (2.27–21.63)**
Seventh	1.31 (0.79–2.19)	1.30 (0.78–2.18)	-	NA

Model 1: adjusted for age category and sex; Model 2: adjusted for age category, sex and pandemic wave of the primary infection; Model 3: adjusted for age category, sex, severity of primary COVID-19, and the time passed from the last dose of vaccine to reinfection (in weeks). aHR—adjusted hazard ratio; 95% CI = 95% confidence interval. Significance levels are given in bold for *p* < 0.05. Ref. = reference category. NA—not applicable.

## Data Availability

The data that support the findings of this study are available from the corresponding author upon reasonable request.

## Supplementary Materials

The following supporting information can be downloaded at: https://www.mdpi.com/article/10.3390/v15112221/s1, Table S1: General characteristics of patients with reinfection based on the year of COVID-19 reinfection registration.
