# Supplementary material for: Epidemiological Characteristics of COVID-19 during Seven Consecutive Epidemiological Waves (2020–2022) in the North Bačka District, Serbia
Source: Viruses. 2023 Nov 7;15(11):2221. doi: 10.3390/v15112221 (PMC10674962; doi:10.3390/v15112221)
Supplement: Supplementary file 1 [file viruses-15-02221-s001.zip › viruses-2642657-supplementary.pdf]

**Supplementary table S1**. General characteristics of patients with reinfection based on the year of COVID-19 reinfection registration.

| | First reinfection (n=3961) | | | Second reinfection (n=106) | | | Total reinfections (n=4067) | | p-value[1] |
|---|---|---|---|---|---|---|---|---|---|
| | 2020 (n=3) | 2021 (n=154) | 2022 (n=3804) | 2020 (n=0) | 2021 (n=0) | 2022 (n=106) | first reinfection (n=3961) | second reinfection (n=106) | |
| **Sex** | | | | | | | | | |
| Male | 0 | 66 (42.86) | 1591 (41.82) | 0 | 0 | 36 (33.96) | 1657 (41.83) | 36 (33.96) | 0.105 |
| Female | 3 (100) | 88 (57.14) | 2213 (58.18) | 0 | 0 | 70 (66.04) | 2304 (58.17) | 70 (66.04) | |
| **Age at infection, years,** mean (SD) | 35 (15.87) | 42.23 (14.78) | 47.53 (15.37) | 0 | 0 | 44.62 (12.94) | 47.32 (15.38) | 44.62 (12.94) | **0.045** |
| **Age category** | | | | | | | | | |
| 0-9 | 0 | 0 | 11 (0.29) | 0 | 0 | 0 | 11 (0.28) | 0 | 0.052 |
| 10-18 | 0 | 7 (4.55) | 81 (2.13) | 0 | 0 | 0 | 88 (2.22) | 0 | |
| 19-29 | 2 (66.67) | 21 (13.64) | 383 (10.07) | 0 | 0 | 12 (11.32) | 406 (10.25) | 12 (11.32) | |
| 30-39 | 0 | 40 (25.97) | 773 (20.32) | 0 | 0 | 28 (26.42) | 813 (20.53) | 28 (26.42) | |
| 40-49 | 0 | 50 (32.47) | 887 (23.32) | 0 | 0 | 31 (29.25) | 937 (23.66) | 31 (29.25) | |
| 50-59 | 1 (33.33) | 14 (9.09) | 754 (19.82) | 0 | 0 | 22 (20.75) | 769 (19.41) | 22 (20.75) | |
| >60 | 0 | 22 (14.29) | 915 (24.05) | 0 | 0 | 13 (12.26) | 937 (23.66) | 13 (12.26) | |
| **Municipality** | | | | | | | | | |
| Bačka Topola | 0 | 9 (5.84) | 199 (5.23) | 0 | 0 | 1 (0.94) | 208 (5.25) | 1 (0.94) | 0.058 |
| Mali Iđoš | 0 | 9 (5.84) | 246 (6.47) | 0 | 0 | 4 (3.78) | 255 (6.44) | 4 (3.78) | |
| Subotica | 3 (100) | 136 (88.31) | 3359 (88.30) | 0 | 0 | 101 (95.28) | 3498 (88.31) | 101 (95.28) | |
| **Occupation** | | | | | | | | | |
| Service provider | 0 | 13 (8.44) | 183 (4.81) | 0 | 0 | 5 (4.72) | 196 (4.95) | 5 (4.72) | 0.086 |
| Healthcare worker | 3 (100) | 27 (17.53) | 287 (7.54) | 0 | 0 | 13 (12.26) | 317 (8.00) | 13 (12.26) | |
| Retirement | 0 | 14 (9.09) | 638 (16.77) | 0 | 0 | 9 (8.49) | 652 (16.46) | 9 (8.49) | |

| | | | | | | | | | |
|---|---|---|---|---|---|---|---|---|---|
| Other | 0 | 100 (64.94) | 2696 (70.87) | 0 | 0 | 79 (74.53) | 2796 (70.59) | 79 (74.53) | |
| **Type of COVID-19 test, n (%)** | | | | | | | | | |
| RT-PCR | 1 (33.33) | 8 (5.19) | 111 (2.92) | 0 | 0 | 4 (3.77) | 120 (3.03) | 4 (3.77) | 0.565 |
| RDT | 2 (66.67) | 146 (94.81) | 3693 (97.08) | 0 | 0 | 102 (96.23) | 3841 (96.97) | 102 (96.23) | |
| **Clinical presentation of COVID-19, n (%)** | | | | | | | | | |
| Asymptomatic | 0 | 9 (5.84) | 57 (1.50) | 0 | 0 | 3 (2.83) | 66 (1.67) | 3 (2.83) | 0.586 |
| Mild | 3 (100) | 141 (91.56) | 3729 (98.03) | 0 | 0 | 103 (97.17) | 3873 (97.78) | 103 (97.17) | |
| Severe | 0 | 4 (2.60) | 17 (0.45) | 0 | 0 | 0 | 21 (0.53) | 0 | |
| Critical | 0 | 0 | 1 (0.03) | 0 | 0 | 0 | 1 (0.03) | 0 | |
| **Comorbidity number, n (%)** | | | | | | | | | |
| None | 3 (100) | 111 (72.08) | 2365 (62.17) | 0 | 0 | 61 (57.55) | 2479 (62.59) | 61 (57.55) | 0.516 |
| One | 0 | 38 (24.68) | 1034 (27.18) | 0 | 0 | 35 (33.02) | 1072 (27.06) | 35 (33.02) | |
| Two | 0 | 5 (3.25) | 315 (8.28) | 0 | 0 | 9 (8.49) | 320 (8.08) | 9 (8.49) | |
| Three or more | 0 | 0 | 90 (2.37) | 0 | 0 | 1 (0.94) | 90 (2.27) | 1 (0.94) | |
| **Type of main comorbidity, n (%)** | | | | | | | | | |
| Obesity | NA | 5 (11.63) | 64 (4.45) | 0 | 0 | 0 | 69 (4.66) | 0 | 0.845 |
| Diabetes | NA | 3 (6.98) | 136 (9.45) | 0 | 0 | 4 (8.89) | 139 (9.38) | 4 (8.89) | |
| Hypertension | NA | 15 (34.88) | 674 (46.84) | 0 | 0 | 25 (55.56) | 689 (46.49) | 25 (55.56) | |
| Malignant disease | NA | 0 | 45 (3.13) | 0 | 0 | 1 (2.22) | 45 (3.04) | 1 (2.22) | |
| Cardiovascular disease | NA | 5 (11.63) | 100 (6.95) | 0 | 0 | 2 (4.44) | 105 (7.09) | 2 (4.44) | |
| Chronic lung disease | NA | 5 (11.63) | 137 (9.52) | 0 | 0 | 4 (8.89) | 142 (9.58) | 4 (8.89) | |
| Other chronic disease or condition | NA | 10 (23.26) | 283 (19.67) | 0 | 0 | 9 (20.00) | 293 (19.77) | 9 (20.00) | |
| **Vaccine at the time of infection** | | | | | | | | | |
| Unvaccinated | 3 (100) | 123 (79.87) | 2794 (73.45) | 0 | 0 | 81 (76.42) | 2920 (73.72) | 81 (76.42) | 0.294 |
| One dose | 0 | 2 (1.3) | 15 (0.39) | 0 | 0 | 0 | 17 (0.43) | 0 | |
| Two doses | 0 | 27 (17.53) | 157 (4.13) | 0 | 0 | 1 (0.94) | 184 (4.65) | 1 (0.94) | |

| | | | | | | | | | |
|---|---|---|---|---|---|---|---|---|---|
| Three doses (booster) | 0 | 2 (1.3) | 838 (22.03) | 0 | 0 | 24 (22.64) | 840 (21.21) | 24 (22.64) | |
| **Type of vaccine in the primary-vaccination (two doses of vaccine) at the time of reinfection** | NA | 31(100) | 1010(100) | 0 | 0 | 25(100) | 1041(100) | 25(100) | NA |
| ChAdOx1 nCoV-19 | NA | 0 | 22 (2.18) | 0 | 0 | 0 | 22 (2.11) | 0 | 0.464 |
| BNT162b2 | NA | 7 (22.58) | 222 (21.98) | 0 | 0 | 4 (16.00) | 229 (22.00) | 4 (16.00) | |
| BBIBP-CorV | NA | 21 (67.74) | 656 (64.95) | 0 | 0 | 16 (64.00) | 677 (65.03) | 16 (64.00) | |
| Gam-COVID-Vac | NA | 3 (9.68) | 110 (10.89) | 0 | 0 | 5 (20.00) | 113 (10.85) | 5 (20.00) | |

[1]Indicators of significance between groups using Pearson's chi-squared test and Fisher's exact test (where appropriate) for categorical and Wilcoxon rank-sum test for continuous variables. Significance levels are given in bold for p<0.05. NA=not applicable. n=number of participants

Explanation: Patients with reinfections were divided based on the years when the reinfections were registered. If the patient had two reinfections, it was countedtwice, i.e. once in primary and the second time in secondary reinfection.
